# Response mechanism of extracellular polymers in the remediation of chromium pollution by carbonate mineralizing bacteria†

**DOI:** 10.1039/d5ra01916h

**Published:** 2025-05-13

**Authors:** Yingying Shen, Huan Cao, Miaomiao Du, Xinfeng Wang, Jia Qin

**Affiliations:** a School of Materials Science and Engineering, Lanzhou University of Technology Lanzhou 730050 China; b School of Optoelectronic Manufacturing, Zhejiang Industry and Trade Vocational College Wenzhou 325002 China Qinjia@zjitc.edu.cn; c Gansu Rare Earth New Material Limited-Liability Company Baiyin 730900 China

## Abstract

This study examines the adaptability of carbonate mineralizing bacteria in Cr(iii)-contaminated environments with varying Cr(iii) concentrations and their response mechanism *via* EPS. Cr(iii) removal efficiency declined with concentrations exceeding 1000 mg L^−1^, while the removal amount continued to rise, indicating strong Cr(iii) tolerance in the bacterium. Analysis of dynamic changes in EPS revealed a significant increase in production, with polysaccharides and proteins playing key roles in Cr(iii) binding. A notable increase in mannose in the monosaccharide composition of EPS suggests its involvement in Cr(iii) binding. Moreover, alterations in the protein secondary structure, such as a reduction in α-helix content and an increase in β-sheet and random coil structures, may enhance EPS interaction with Cr(iii). These findings demonstrate that EPS contributes to heavy metal remediation not only through its polysaccharide components but also through changes in protein structure, offering a new theoretical foundation for Cr(iii) bioremediation.

## Abstract 2

This study examines the adaptability of carbonate mineralizing bacteria in Cr(iii)-contaminated environments with varying Cr(iii) concentrations and their response mechanism *via* EPS.

## Graphical abstract

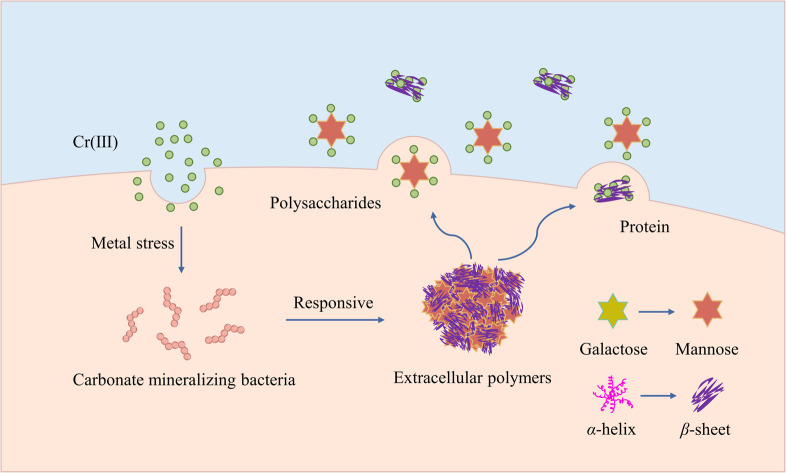

## 1. Introduction

The rapid pace of global industrialization has significantly increased the prevalence of heavy metal pollution, positioning it as a critical issue in the fields of environmental science and ecological restoration.^1^ Trivalent chromium (Cr(iii)), a persistent and ecotoxic heavy metal, is frequently found in high concentrations in industrial effluents, particularly from sectors such as electroplating, leather tanning, and steel production.^2^ Although Cr(iii) is comparatively less toxic to the environment and organisms at low concentrations, it can still pose severe risks to aquatic ecosystems, soil quality, and human health through bioaccumulation in the food chain when present in elevated levels.^3^ Consequently, the remediation of chromium pollution has become an urgent concern within environmental protection efforts.

Bioremediation techniques exploit the natural metabolic capabilities of microorganisms to degrade or transform metal pollutants, offering a sustainable solution to environmental contamination. These methods are particularly advantageous due to their low cost, minimal secondary pollution, and significant environmental adaptability.^4^ Over the past few decades, microbial-induced carbonate precipitation (MICP) has gained prominence as a key mechanism for immobilizing heavy metals. In the MICP process, microorganisms facilitate the conversion of dissolved metal ions into insoluble mineral forms, effectively reducing both the bioavailability and toxicity of these metals.^5^ This biogeochemical process has proven particularly effective in mitigating the environmental risks posed by heavy metals such as chromium. Despite the growing body of research on MICP and its capacity for metal remediation, the exact role of extracellular polymers (EPS) in this process remains underexplored. EPS, which are polymers secreted by microorganisms during their growth, can significantly influence the adsorption, immobilization, and transformation of metal ions.^6^ These polymers can form stable complexes with metal ions, enhancing the microorganisms' ability to sequester heavy metals from the environment. As such, a deeper understanding of the mechanisms by which EPS contributes to MICP is crucial for optimizing bioremediation strategies and advancing the development of more effective and targeted metal remediation technologies.

EPS are high-molecular-weight compounds secreted by microorganisms into the surrounding environment. These complex macromolecules are primarily composed of polysaccharides, proteins, nucleic acids, and a minor fraction of lipids.^7^ One of the fundamental roles of EPS is to safeguard microorganisms from environmental stress, particularly from toxic substances such as metal ions. This protection is achieved through complexation or adsorption of metal ions, effectively reducing the concentration of free ions and mitigating their toxic effects on microbial cells.^8^ In metal-contaminated environments, microorganisms often upregulate EPS production, especially in response to heavy metal exposure, where secretion of EPS typically increases significantly.^9^ Among the diverse components of EPS, the polysaccharide fraction plays a central role due to its strong metal-ion adsorption capacity and its ability to immobilize metal ions through complexation, thus reducing their bioavailability.^10^ Functional groups, such as hydroxyl groups, present in the polysaccharides, facilitate interactions with metal ions, further diminishing their reactivity and promoting their immobilization.^11^ Additionally, the stability of EPS in aquatic environments and its ability to concentrate high levels of metal ions make it an essential component in the remediation of heavy metal pollution. The protein fraction of EPS also contributes significantly to metal ion binding. Proteins in EPS are rich in amino acid residues, such as cysteines, which contain sulfur groups capable of coordinating with metal ions. This ligand-binding capacity further enhances the adsorption of metals, providing an additional mechanism by which EPS assists in heavy metal sequestration.^12^

In the field of microbial remediation, EPS produced by various microorganisms play critical roles in the removal of metals from contaminated environments. For instance, glucose-induced biofilm formation has been shown to stimulate EPS production in *Bacillus cereus* KPWP1, enhancing the bacterium's ability to adapt to environmental pollutants, particularly in heavy metal-contaminated settings.^13^*Virgibacillus* sp. has demonstrated remarkable efficacy in metal immobilization. The EPS it produces forms stable complexes with metal ions, including Cd^2+^, Cu^2+^, Ni^2+^, and Zn^2+^, thereby promoting mineral deposition. Specific functional groups, such as carboxyl and phosphate groups, present in the secreted EPS interact with metal ions, significantly influencing the immobilization process of these metals.^14^ Additionally, *Graesiella*, a thermophilic green alga, produces EPS that exhibit not only biological activities such as antioxidant and antimicrobial properties but also strong emulsification and flocculation characteristics. This EPS is effective in adsorbing and removing metal ions from aqueous environments, including Zn^2+^ and Cd^2+^.^15^ Furthermore, EPS produced by sulfate-reducing bacteria display a remarkable calcium-binding capacity, playing a key role in the formation of carbonate minerals by influencing their precipitation.^16^ These findings collectively highlight the diverse and complex roles of EPS, underscoring their significant potential in a wide range of applications for metal pollution remediation.

This study aims to investigate the mechanisms underlying the response of a novel carbonate mineralizing bacterium to metal contamination, with a particular focus on the role of EPS under Cr(iii) stress. The research will specifically analyze how varying concentrations of Cr(iii) influence the monosaccharide composition and protein secondary structure of EPS, and how these changes contribute to the bacterium's adaptation to different levels of chromium stress. By examining the dynamic alterations in EPS, the study hypothesizes that it will uncover the pivotal role of EPS in the bioremediation of Cr(iii) by carbonate mineralizing bacteria. This research will provide insights into how EPS facilitates the detoxification and immobilization of Cr(iii), thus contributing to the development of more effective bioremediation strategies.

## 2. Materials and methods

### 2.1 Materials

A novel carbonate mineralizing bacterium, *Bacillus* sp., was employed in this study. This bacterium was obtained from Weiyuan Biotechnology (Guangzhou, China). Beef paste and peptone, essential for bacterial growth, were procured from Beijing Auboxing Biotechnology (Beijing, China). The staining solution, Thomas Brilliant Blue G-250, and bovine serum proteins were sourced from Weibokang Biotechnology (Fuzhou, China). Anhydrous HCl methanol was procured from Guangdong Qiyuan Pharmaceutical and Chemical (Guangdong, China). Concentrated sulfuric acid, phenol, and glucose were analytically pure and obtained from Shanghai Aladdin Biochemical Science and Technology (Shanghai, China). The analytically pure substances utilized in this study include CrCl_3_·6H_2_O, CaCl_2_, KBr, ethanol, myo-inositol, pyridine, hexamethyldisilazane, and trichloromethylsilane, all of which were obtained from Shanghai McLean Biochemical Science and Technology (Shanghai, China).

### 2.2 Growth of carbonate mineralizing bacteria

A total of 2.0 g of bacterial powder was weighed and introduced into a natural liquid medium, which had been pre-mixed with 1.0 g of beef paste, 0.6 g of peptone, and 200 mL of ultrapure water. The optimal culture conditions had been previously determined,^17^ with the initial pH of the medium adjusted to 7.0. The bacteria were then cultured in a thermostatic shaking incubator (THZ-98AB, Shanghai Yihang, China) at 25 °C and 180 rpm for 48 h. Upon completion of the culture period, the resulting bacterial culture was stored at 4 °C in a refrigerator for future use.

### 2.3 Cr(iii) removal experiments

A 100 mL volume of bacterial solution was mixed with an equal volume of Cr(iii) solution, maintaining a 1 : 1 ratio. The concentrations of Cr(iii) used in the experiment were 0, 500, 1000, 1500, and 3000 mg L^−1^. The initial pH of the mixture was adjusted to 7.0, and 1.0 g of CaCl_2_ was added. The combined solution was then incubated in an intelligent biochemical incubator (SPX-150, Shanghai YiXi, China) at 25 °C. To monitor bacterial growth in the presence of Cr(iii), samples were collected at 0, 6, 12, 24, 36, and 48 h, and the OD_600_ values were measured using a UV-visible spectrophotometer (UV-1800PC-DS2, Shanghai Mepda, China) at 600 nm. The OD_600_ value is commonly used as an indirect measure of microbial growth activity. After 48 h of incubation, the reaction mixture was analyzed. The filtrate of the reaction solution, passed through a 0.22 μm filter, was then analyzed for residual Cr(iii) content using flame atomic absorption spectrometry (AAS) and diphenylurea (DPC).^18^ The amount of Cr(iii) removed and the removal efficiency were subsequently calculated based on the results of these analyses.

### 2.4 EPS recovery

After 48 h of incubation at varying Cr(iii) concentrations, the reaction solutions were subjected to centrifugation using a high-speed cryo-centrifuge (H1750R, Hunan Xiangyi, China) at 4 °C and 8000 rpm for 30 min. Following centrifugation, the resultant filtrate was passed through a 0.22 μm sterile filter to isolate the EPS fraction in the dissolved state.^19^ The filtrate was then mixed with pre-prepared cold ethanol at a ratio of 1 : 3 and allowed to precipitate at −20 °C for 48 h. Dialysis purification was subsequently performed using a dialysis membrane with a molecular weight cutoff of 10 kDa, with the solution stirred in an ice-water bath. The purified dialysate was centrifuged at 10 000 rpm for 30 min, yielding a precipitate that was collected as EPS.^20^ The EPS sample was then transferred and dried to a vacuum freeze-dryer (Scientz-10 YG, Ningbo Xinzhi, China) for 30 h. The dried EPS was weighed, and the samples were labeled according to the Cr(iii) concentrations used, designated as 0Cr, 500Cr, 1000Cr, 1500Cr, and 3000Cr.

### 2.5 Determination of polysaccharide and protein content in EPS

The phenol–sulfuric acid method was used to quantify the polysaccharide content in EPS. This method is based on the reaction between phenol and monosaccharide molecules, such as glucose, in the presence of concentrated sulfuric acid at elevated temperatures, which results in the formation of organic sugar aldehydes and their derivatives. These compounds exhibit characteristic color changes, and their absorbance at a wavelength of 490 nm shows a linear relationship with the polysaccharide concentration.^21^ To prepare the standard solutions, 10 mg of glucose was accurately weighed and dissolved in 100 mL of water, resulting in a 100 mg L^−1^ glucose stock solution. This stock solution was then used to prepare glucose standard solutions at concentrations of 0, 10, 20, 30, 40, and 50 mg L^−1^. One milliliter of each standard solution was transferred to a colorimetric tube, followed by the addition of 1 mL of 6% phenol and 5 mL of concentrated sulfuric acid. The tube was heated in a water bath at 95 °C for 10 min, after which it was removed and allowed to cool for 30 min. The absorbances of the samples were measured at 490 nm, and a glucose standard curve was constructed. The absorbances of appropriately diluted EPS samples were then measured, and the polysaccharide content was calculated using the standard curve (Fig. S1†).

The protein content in EPS was determined using the Coomassie Brilliant Blue method, which involves the binding of Coomassie Brilliant Blue G-250 dye to proteins. This binding leads to a shift in the maximum absorption wavelength of the dye from 465 nm to 595 nm. The absorbance of the color-developing solution at 595 nm increases linearly with the protein concentration.^22^ The protein concentration of the stock solution was accurately measured at 100 mg L^−1^. This stock solution was then used to prepare glucose standard solutions at concentrations of 0, 10, 20, 30, 40, and 50 mg L^−1^. One milliliter of each standard solution was transferred to a colorimetric tube, followed by the addition of 1 mL of 6% phenol and 5 mL of concentrated sulfuric acid. The tube was heated in a water bath at 95 °C for 10 min, after which it was removed and allowed to cool for 30 min. The absorbances of the samples were measured at 490 nm, and a glucose standard curve was constructed. The absorbances of appropriately diluted EPS samples were then measured, and the protein content was calculated using the standard curve (Fig. S2†).

### 2.6 Scanning electron microscopy (SEM) imaging

The morphology and structure of EPS were observed using a field emission scanning electron microscope (SU8010, Hitachi, Japan) at high magnification under a stabilized voltage of 3.0 kV. Simultaneously, the EPS samples underwent energy dispersive X-ray spectroscopy (EDS) to analyze their elemental composition. Due to the non-conductive nature of EPS, the samples were treated with platinum sputtering using a high-vacuum ion sputtering apparatus (EM ACE600, Leica, Germany) to enhance their conductivity prior to observation.

### 2.7 Fourier infrared spectroscopy (FT-IR) and protein secondary structure

The prepared EPS samples were mixed with potassium bromide (KBr) at a 1 : 100 ratio and analyzed using a Fourier transform infrared spectrometer (FTIR-650, Tianjin Gangdong, China) within the wavelength range of 4000–400 cm^−1^. This analysis was conducted to characterize the molecular structure, including chemical bonds and functional groups, present in the EPS. Simultaneously, the infrared absorption peaks in the amide I region (1700–1600 cm^−1^) were isolated and fitted using PeakFit software to analyze the secondary structure of proteins in the EPS.

### 2.8 Determination of EPS monosaccharide composition by gas chromatography-mass spectrometry (GC-MS)

The EPS sample was dissolved in 1.5 mL of 1 mol L^−1^ anhydrous HCl methanol and placed in a water bath at 85 °C for 18 h to ensure complete hydrolysis of the monosaccharides in the EPS to their corresponding glycosides. After cooling, 0.2 mL of an internal standard solution (inositol, dissolved in pyridine at a concentration of 100 mg L^−1^) was added. The solution was then dried using a circular water bath nitrogen blower (JC-WD, Qingdao Polytron, China) at 40 °C. Subsequently, 2 mL of a solution containing pyridine, hexamethyldisilazane, and trimethylchlorosilane (10 : 2 : 1) was added to the dried sample and incubated in a water bath at 85 °C for 30 min to complete the trimethylsilylation of glycosides (TMS).^23^ Finally, the monosaccharide composition of EPS was analyzed using a gas chromatography-mass spectrometer (GCMS-QP2020NX, Shimadzu, Japan). Chromatographic analysis was performed on an HP-5 column (30 m × 0.25 mm × 0.25 μm) with a helium flow rate set at 1.5 mL min^−1^, an inlet temperature of 280 °C, and a split ratio of 1 : 10. The heating procedure was as follows: the initial temperature was set at 130 °C and held for 2 min, then heated to 280 °C at a rate of 2 °C min^−1^ and held for 7 min. The ion source temperature was set at 230 °C, the energy of the electron bombardment (EI) source was 70 eV, and the injection volume was 1 μL. Three parallel determinations were performed for each sample.

## 3. Results and discussion

### 3.1 Removal of Cr(iii) and EPS components by carbonate mineralizing bacteria

**Fig. 1:**
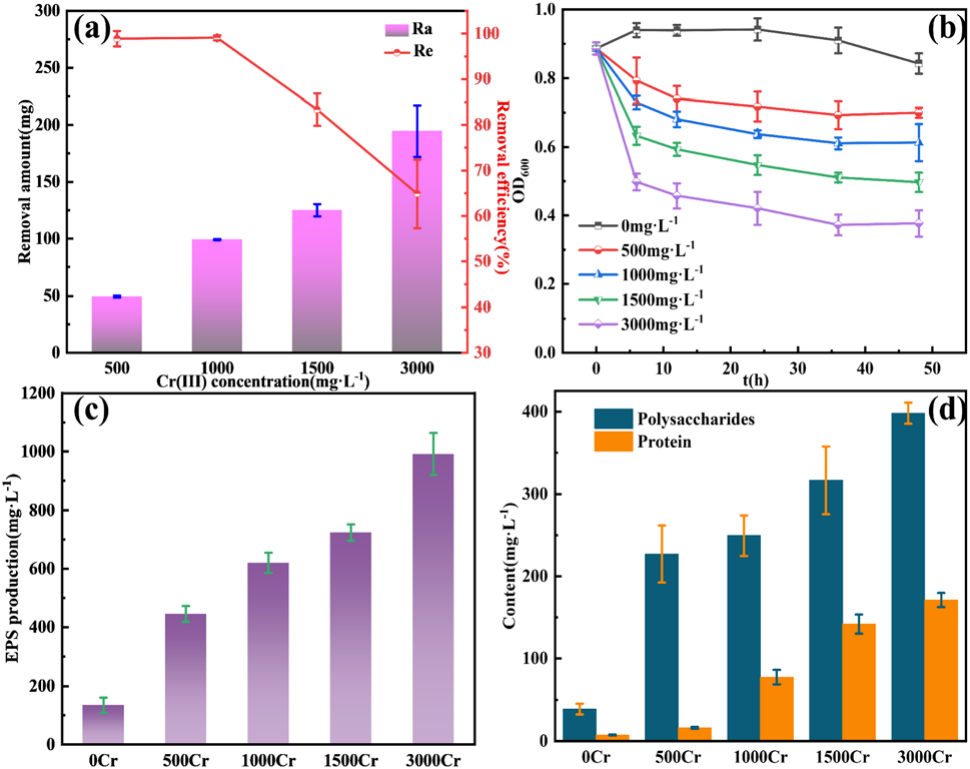
Add different Cr(iii) concentrations of (a) removal amount (Ra) and removal efficiency (Re); (b) OD_600_; (c) EPS production; (d) polysaccharides and protein content in EPS.

As shown in Fig. 1a, the removal amount and removal efficiency for different concentrations of Cr(iii) are presented. At Cr(iii) concentrations of 500 and 1000 mg L^−1^, the removal efficiencies were nearly 100%, 98.88%, and 99.08%, respectively. However, when the Cr(iii) concentration was increased to 1500 mg L^−1^, the removal efficiency significantly decreased to 83.34%, and at 3000 mg L^−1^, the removal efficiency further dropped to 64.85%. Despite this decline, the removal amount showed an upward trend with increasing Cr(iii) concentrations, suggesting that the bacterium maintained its effectiveness in Cr(iii) removal even at a concentration of 3000 mg L^−1^, demonstrating tolerance to high Cr(iii) concentrations. Fig. 1b illustrates bacterial growth after Cr(iii) addition. Within 6 h post-Cr(iii) addition, a rapid decrease in bacterial activity was observed, which can be attributed to the stressful effects of Cr(iii). As the incubation period extended, the decline in bacterial activity slowed, and slight recovery in activity was observed between 36 and 48 h of continuous incubation. This recovery suggests that the bacterium possesses some degree of adaptability and tolerance to Cr(iii) stress. As shown in Fig. 1c, the addition of Cr(iii) led to a significant increase in EPS production, suggesting that the bacterium synthesizes EPS in response to Cr(iii) stress. This observation, coupled with the changes in the OD_600_ of the bacterium, suggests that EPS plays a crucial role in the bacterium's adaptation to Cr(iii) stress. The contents of polysaccharides and proteins in EPS are presented in Fig. 1d. The addition of Cr(iii) significantly increased both protein and polysaccharide contents in EPS, with polysaccharide changes being more significant than protein changes, indicating that polysaccharides played a more prominent role in EPS in response to Cr(iii) stress.

### 3.2 SEM

**Fig. 2:**
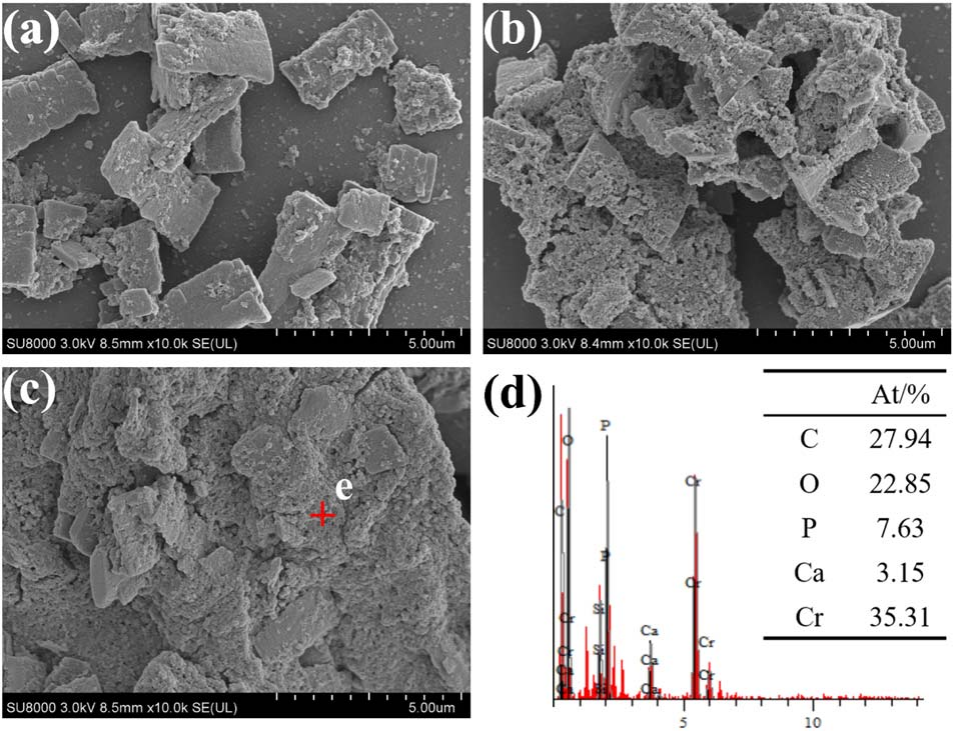
SEM images of EPS with different Cr(iii) concentrations. (a) 0Cr; (b) 1000Cr; (c) 3000Cr; (d) energy spectrum analysis of point e.

As illustrated in Fig. 2a, a scanning electron micrograph of EPS without added Cr(iii) reveals a rectangular irregular block structure with a relatively smooth surface. Fig. 2b presents a photograph of EPS following the incorporation of 1000 mg L^−1^ Cr(iii), which exhibits a marked increase in surface looseness and porosity. EPS is composed of polysaccharides and proteins, and its surface contains a high concentration of hydrophilic functional groups, which serve as potential binding sites for trapping organic molecules and metal ions. These characteristics provide favorable conditions for resisting the coercive effects of Cr(iii) ions in water.^24^ This, in turn, helps to maintain the activity of carbonate mineralizing bacteria. Fig. 2c shows a photograph of the EPS after the addition of 3000 mg L^−1^ Cr(iii), where the surface is almost completely covered by a more loose and uniformly distributed porous structure compared to Fig. 2b. Fig. 2d presents the energy spectrum analysis of point e in Fig. 2c, indicating that Cr (35.31%), C (27.94%), and O (22.85%) are the predominant elements. It has been demonstrated that the abundance of reactive functional groups on the surface of EPS leads to a high affinity for metal ions, thereby promoting the formation of stable complexes with metal ions, such as a double-toothed bridge structure.^25^ The substantial presence of C and O suggests that polysaccharides and protein molecules are the primary components of the EPS, in addition to a modest amount of P, which indicates the presence of phosphorylated groups in the EPS.^26^ Consequently, the outcomes of elemental analyses imply that EPS may have engaged in interactions with Cr(iii), resulting in the formation of stable metal–ligand complexes. These complexes have been shown to enhance the resistance of carbonate-mineralizing bacteria to Cr(iii).

### 3.3 FT-IR

**Fig. 3:**
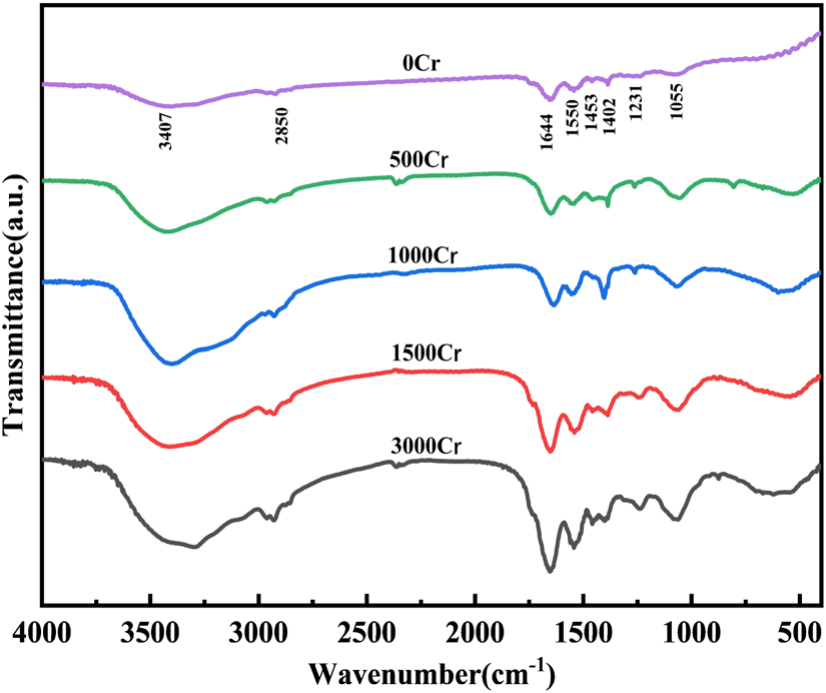
FT-IR of EPS produced by different Cr(iii) concentrations.

In this study, the chemical composition of EPS and its alterations in Cr(iii)-contaminated environments were analyzed using FT-IR spectra (Fig. 3). The FT-IR spectra provide significant molecular insights, particularly in the analysis of EPS's protein and polysaccharide composition and the mechanism of interaction with Cr(iii).^27^ Below is a comprehensive analysis of the spectral data:

The characteristic peaks of stretching vibrations such as –OH and N–H at 3407 cm^−1^, which can form hydrogen bonds, are attributed to the hydroxyl groups in the polysaccharide and protein structures, as well as the amine groups in the proteins. This suggests that the extracellular polymer contains both polysaccharide and protein fractions.^28^ The peaks at 1700–1500 cm^−1^ and 1300–900 cm^−1^ correspond to protein and polysaccharide components, respectively, confirming the presence of these fractions in the polymer.^29^ As Cr(iii) concentration increased, the intensity of the polysaccharide and protein absorption peaks exhibited a general upward trend, indicating that the polysaccharide and protein contents in EPS increase under Cr(iii) stress. This finding is consistent with previous analyses.

The absorption peak at 2850 cm^−1^ corresponds to the stretching vibration of methyl or methylene groups in saturated hydrocarbon chains, while the 1453 cm^−1^ peak reflects the shear vibration of methylene groups in the polymer, indicating the presence of lipid or hydrophobic groups in EPS.^30^ The peak at 1644 cm^−1^ represents the characteristic amide I absorption of proteins, linked to the C=O stretching and N–H bending vibrations of the peptide bond, confirming protein structure.^31^ The 1550 cm^−1^ peak is the amide II absorption, associated with N–H bending and C–N stretching vibrations.^32^ The interaction between peptide bonds and Cr(iii) ions increases with Cr(iii) concentration, as seen in the changes of amide I and II peaks. A symmetric COO– vibration appeared at 1402 cm^−1^,^33^ with peak intensity initially increasing and then decreasing, suggesting the involvement of carboxylate groups in Cr(iii) complexation. The peak at 1231 cm^−1^, attributed to the P=O stretching in phosphorylated proteins or nucleic acids,^34^ showed a slight intensity increase, indicating some disruption in the carbonate mineralizing bacterium, but also suggesting Cr(iii) tolerance. Additionally, the C–O–C stretching vibration at 1055 cm^−1^ in the polysaccharide fraction was enhanced after Cr(iii) addition,^35^ implying that the high polysaccharide content in EPS may aid in Cr(iii) immobilization and segregation.

In summary, the results of infrared spectroscopic analysis indicated that the polysaccharide and protein components in the extracellular polymer changed under Cr(iii) stress, especially in the enhanced response of functional groups (carboxyl groups, phosphate groups, *etc.*) bound to Cr(iii) ions. These findings underscore the pivotal function of EPS in Cr(iii) adsorption, complexation, and bioremediation processes.

### 3.4 GC-MS

**Fig. 4:**
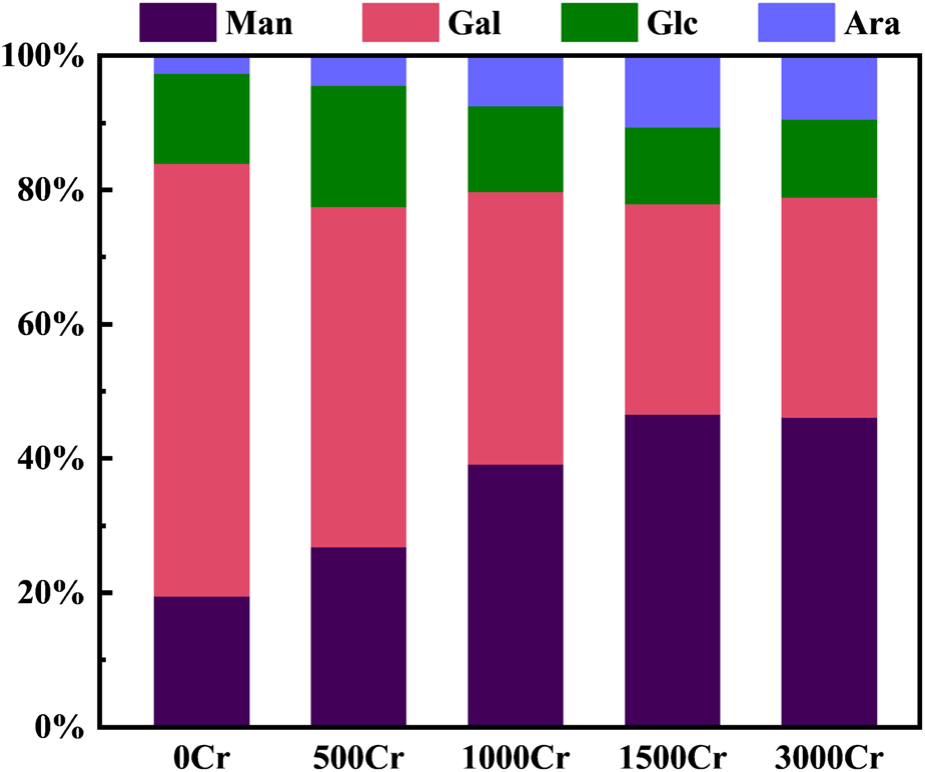
Monosaccharide composition of EPS produced by different Cr(iii) concentrations.

A comprehensive analysis of the monosaccharide composition of EPS from the carbonate mineralizing bacterium was conducted using GC-MS (Fig. S3–S7†). This analysis identified four predominant monosaccharide fractions, as shown in Table S1:† mannose (Man), galactose (Gal), glucose (Glc), and arabinose (Ara). These monosaccharides have been previously reported in related studies.^36^ In Fig. 4, mannose and galactose consistently accounted for approximately 80% of the total monosaccharides under varying Cr(iii) concentrations, highlighting their prevalence as the primary monosaccharide components of EPS.

In the absence of Cr(iii), the galactose content in EPS was 64.48%. As the Cr(iii) concentration increased, galactose content decreased significantly, while mannose content increased. Additionally, arabinose content exhibited a slight increase, whereas glucose content remained relatively stable. These findings suggest that the monosaccharide composition of EPS shifts from galactose to mannose and arabinose as Cr(iii) concentration rises. This indicates that mannose and arabinose may play crucial roles in the interaction between EPS and Cr(iii) in EPS produced by carbonate mineralizing bacteria under Cr(iii) stress. The observed increase in mannose content suggests that mannose may serve as the primary target for EPS binding to Cr(iii),^37^ potentially contributing to the stress resistance mechanism of carbonate mineralizing bacteria. In contrast, glucose content remained stable, with no significant changes. Previous studies have suggested that glucose functions as a stabilizing monomer in the interaction of EPS with heavy metals,^38^ which may explain the stability of glucose levels in EPS under these conditions.

The monosaccharide composition of EPS exhibited a dynamic response to increasing Cr(iii) concentration. A significant increase in mannose was observed, suggesting that it may play a crucial role in the binding and mineralization of EPS with Cr(iii) and may be the primary target molecule for Cr(iii) ion binding. This study provides a novel perspective on the response of carbonate mineralizing bacteria to metal ion stress through extracellular polysaccharides.

### 3.5 Protein secondary structure

**Fig. 5:**
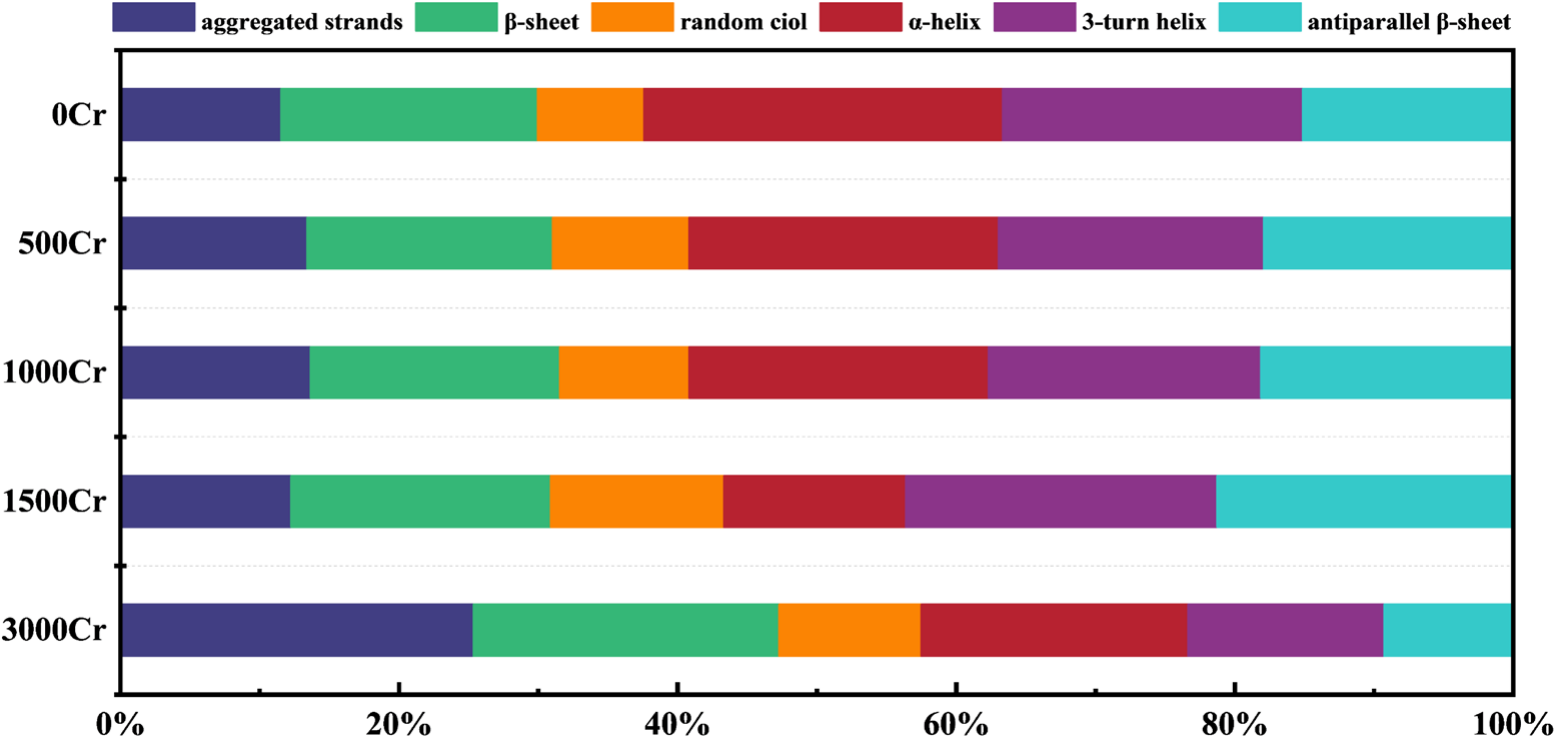
Changes in protein secondary structure in EPS produced by the addition of different Cr(iii) concentrations.

**Fig. 6:**
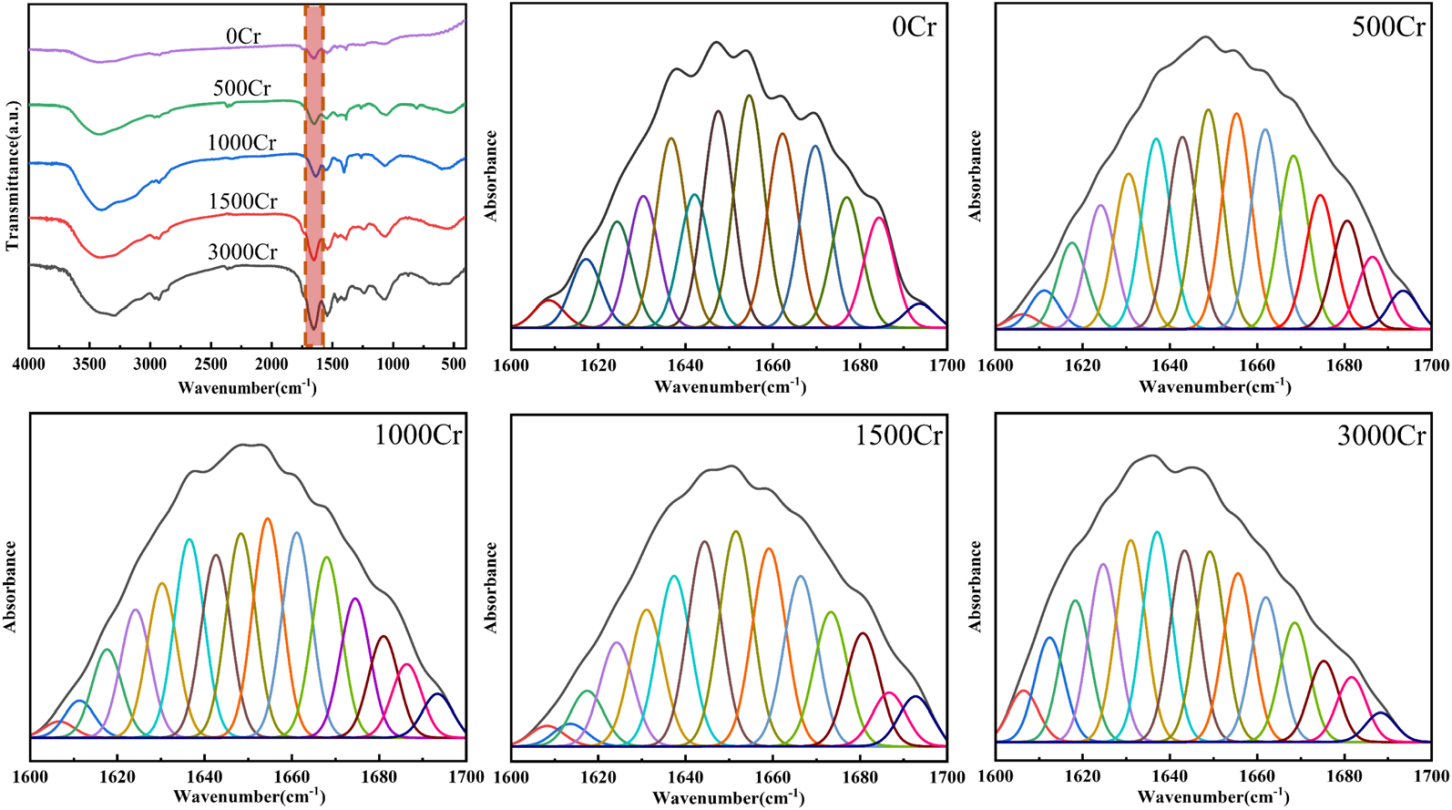
Amide region (1700–1600 cm^−1^) of the peakfit-fitted infrared spectrum.

Proteins are indispensable components of EPS and play a crucial role in the interaction between EPS and metal ions.^39^ To thoroughly examine the effect of Cr(iii) on the secondary structure of proteins in EPS, the data from the amide I region (1700–1600 cm^−1^) in FT-IR were split-peak fitted (Fig. 6), and the fitted peaks were attributed to the six major protein secondary structures (Table 1 and Fig. 5).^40^ The findings revealed a concentration-dependent increase in the proportion of proteins with antiparallel β-sheet and random ciol structures, accompanied by a concurrent decrease in the proportion of α-helical proteins, while the contents of polymerized chain, β-sheet, and 3-turn helix proteins remained relatively stable. It has been previously suggested that a lower ratio of α-helix/(β-sheet + random ciol) implies that the more loosely structured the proteins are, the more easily their active sites are exposed,^41^ which is more favorable for the interaction of EPS with Cr(iii). This structural loosening may enhance the adaptability of EPS by increasing the accessibility of potential binding sites. However, it is important to note that these structural changes could also indicate protein denaturation under high Cr(iii) concentrations, potentially compromising the functional integrity of EPS and affecting the physiological response of the bacteria. The observed loosening trend is consistent with findings from previous studies using SEM, but further research is required to determine whether these changes represent adaptive restructuring or result from protein denaturation. The present study further confirmed the regulation of EPS protein structure by Cr(iii) ions. Therefore, the dynamic changes in the secondary structure of EPS proteins may represent an important mechanism for their protective role in Cr(iii)-contaminated environments.

**Table 1:** Classification of protein secondary structures in EPS

Secondary structures	Wavenumber (cm^−1^)	At (%)				
		0Cr	500Cr	1000Cr	1500Cr	3000Cr
Aggregated strands	1625–1610	11.54	13.39	13.64	12.26	25.32
β-Sheet	1640–1630	18.41	17.64	17.91	18.63	21.96
Random ciol	1645–1640	7.64	9.80	9.27	12.44	10.21
α-Helix	1657–1648	25.74	22.20	21.49	13.06	19.15
3-Turn helix	1666–1659	21.55	19.02	19.57	22.35	14.06
Antiparallel β-sheet	1698–1680	15.14	17.94	18.13	21.27	9.30

### 3.6 EPS response mechanism

**Fig. 7:**
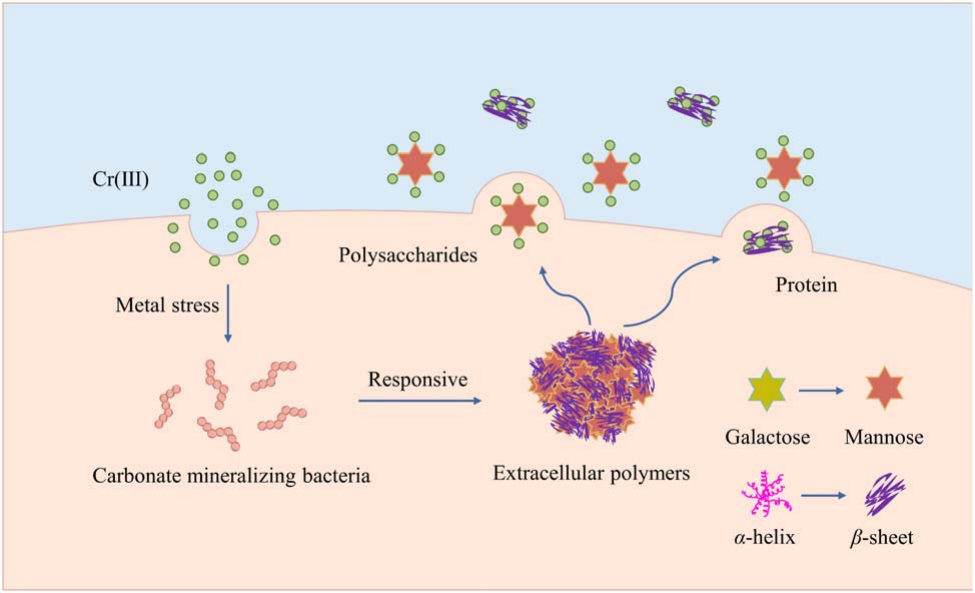
Reaction mechanism of EPS to Cr(iii).

The mechanism by which EPS responds to Cr(iii) is depicted in Fig. 7. In response to the metal stress induced by Cr(iii), carbonate-mineralized bacteria secrete a significant amount of EPS to ensure their protection and preserve their biological activity. The polysaccharide components and protein structure of EPS undergo structural transformations, where monosaccharides are converted from galactose to mannose. This modification of monosaccharides enables EPS to interact more effectively with Cr(iii). The protein structure showed a reduction in α-helix content, accompanied by an increase in β-sheet and random coil structures. This structural alteration increased the porosity of the EPS surface, thereby enhancing its interaction with functional groups, including hydroxyl groups, on the EPS surface and Cr(iii). Consequently, this enhancement improved the resistance and repair capabilities of carbonate-mineralized bacteria. While the present study provided valuable insights into the role of EPS in Cr(iii)-contaminated environments, it also has limitations, particularly due to the experiments being conducted under single metal ion contamination conditions. Future research should expand the investigation to include multi-metal environments to better understand EPS's broader response mechanisms. Additionally, further exploration is needed to identify the specific molecular interactions between EPS and metal ions.

## 4. Conclusions

This study comprehensively investigated the adaptation mechanisms employed by carbonate mineralizing bacteria in Cr(iii)-contaminated environments, with particular emphasis on the role of EPS in Cr(iii) remediation. The findings demonstrated:

(1) EPS composition: as Cr(iii) concentration increased, EPS production in carbonate-mineralizing bacteria rose, particularly polysaccharides and proteins. EPS plays a key role in bacterial adaptation to Cr(iii), enhancing tolerance and repair through binding with Cr(iii).

(2) Structural changes of EPS: EPS surface structure altered, becoming looser and more porous, enhancing its metal ion adsorption capacity. Hydrophilic functional groups in EPS aid in Cr(iii) capture and reduce toxicity by forming stabilizing complexes with metal ions.

(3) Changes in polysaccharides and protein structure: increased mannose content highlighted it as the target sugar for EPS interaction with Cr(iii). The decrease in α-helix as well as increase in β-sheet and random ciol structures facilitated EPS interaction with Cr(iii), enhancing its role in remediation.

## Data availability

Data for this article is available at Science Data Bank at https://doi.org/10.57760/sciencedb.22309.

## Author contributions

Y. S.: conceptualization, funding acquisition, project administration, resources, writing – review and editing. H. C.: data curation, formal analysis, writing – original draft. M. D.: software, investigation. X. W.: project administration, validation. J. Q.: funding acquisition, resources, visualization, supervision.

## Conflicts of interest

There are no conflicts to declare.

## Supplementary Material

RA-015-D5RA01916H-s001
