# Supplementary material for: Response mechanism of extracellular polymers in the remediation of chromium pollution by carbonate mineralizing bacteria
Source: RSC Adv. 2025 May 13;15(18):14227–34. doi: 10.1039/d5ra01916h (PMC12070257; doi:10.1039/d5ra01916h)
Supplement: RA-015-D5RA01916H-s001 [file RA-015-D5RA01916H-s001.pdf]

Supplementary Information (SI) for RSC Advances.
This journal is © The Royal Society of Chemistry 2025

Supplementary material

Response Mechanism of Extracellular Polymers in the Remediation of Chromium Pollution by Carbonate Mineralizing Bacteria

Yingying Shen[a], Huan Cao[a], Miaomiao Du[b], Xinfeng Wang[c], Jia Qin[b,*]

[a] *School of Materials Science and Engineering, Lanzhou University of Technology, Lanzhou 730050, China*

[b] *School of Optoelectronic Manufacturing, Zhejiang Industry and Trade Vocational College, Wenzhou 325002, China*

[c] *Gansu Rare Earth New Material Limited-Liability Company, Baiyin 730900, China*

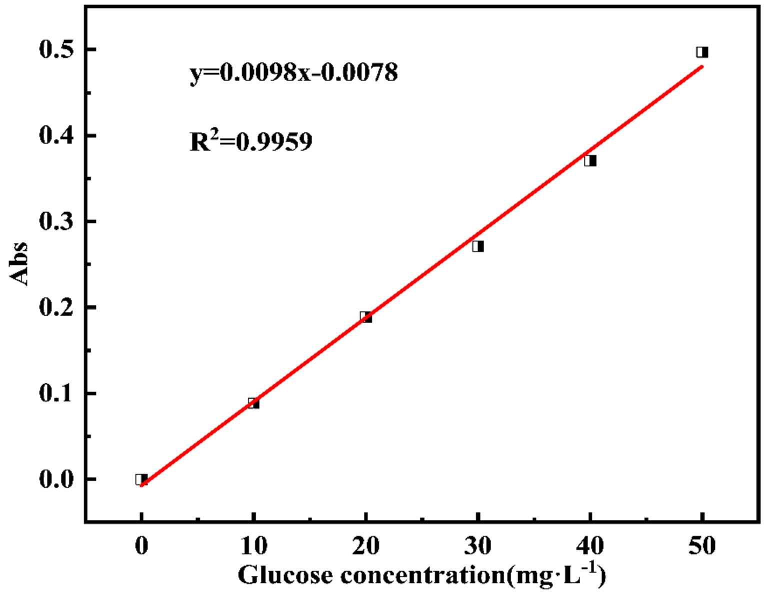

Fig.S1. Standard curves for Glucose.

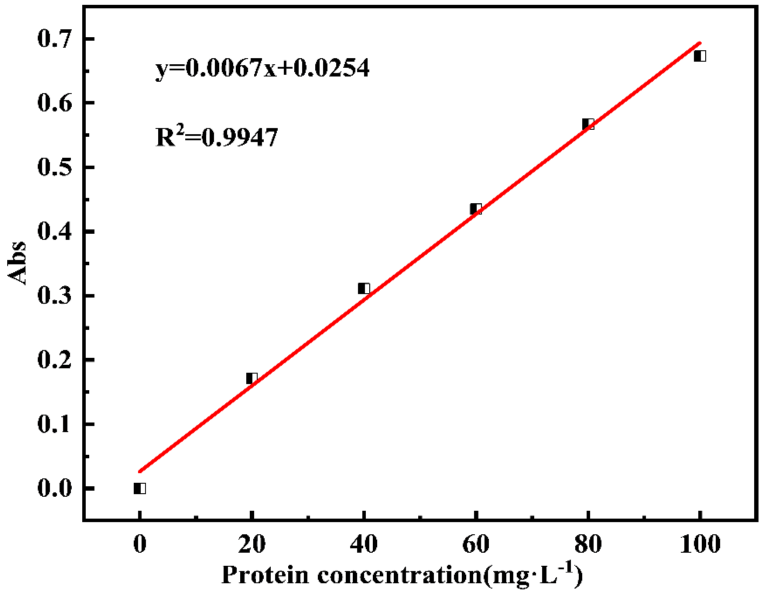

Fig.S2. Standard curves for Protein.

GC-MS

The GC chromatogram of EPS is displayed in Figures S3-S7, with peaks 1-7 designated as follows:

(1) β-L-l,2,3,4-tetrakis-O-(trimethylsilyl)-Arabinpyranose.

(2) β-D-l,2,3,5,6-pentakis-O-(trimethylsilyl)-Galactofuranose.

(3) α-D-methyl-2,3,4,6-tetrakis-O-(trimethylsilyl)-Glucopyranoside.

(4) α-D-1,2,3,4,6-pentakis-O-(trimethylsilyl)-Mannopyranoside.

(5) β-D- methyl-2,3,4,6-tetrakis-O-(trimethylsilyl)-Galactopyranoside.

(6) α-D-1,2,3,4,6-pentakis-O-(trimethylsilyl)-Galactopyranoside.

(7) Myo-Inositol-6TMS.

Table S1 Classification of monose in EPS

Glycosyl residue	At(%)				
	0Cr	500Cr	1000Cr	1500Cr	3000Cr
Mannose(Man)	19.50	26.88	39.17	46.64	46.15
Galactose(Gal)	64.48	50.68	40.61	31.32	32.81
Glucose(Glc)	13.37	18.00	12.81	11.46	11.66
Arabinose(Ara)	2.65	4.44	7.40	10.58	9.38

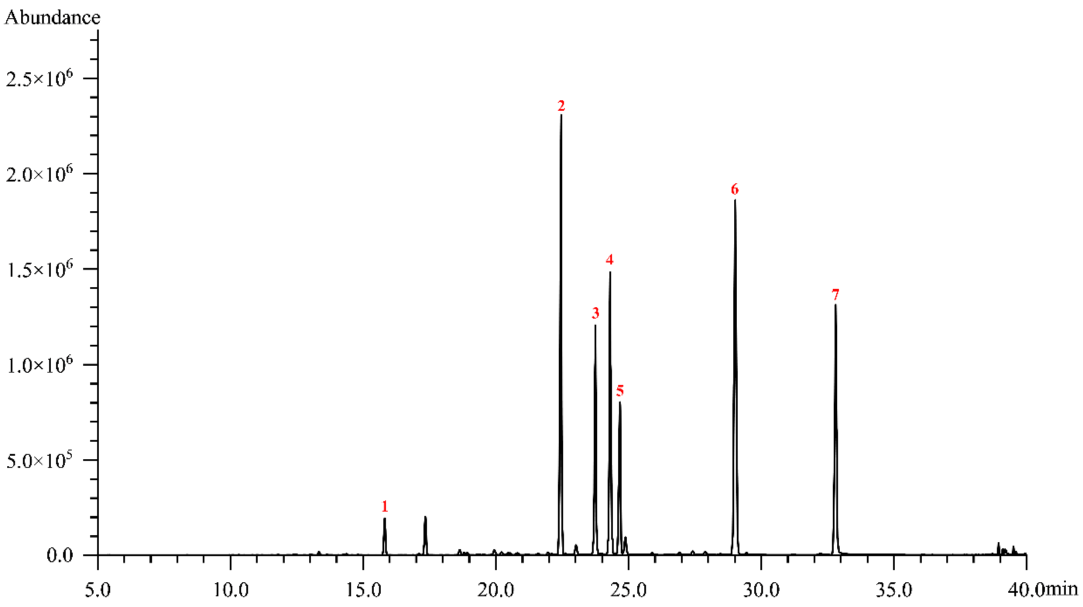

Fig. S3. GC chromatogram obtained for sample 0Cr

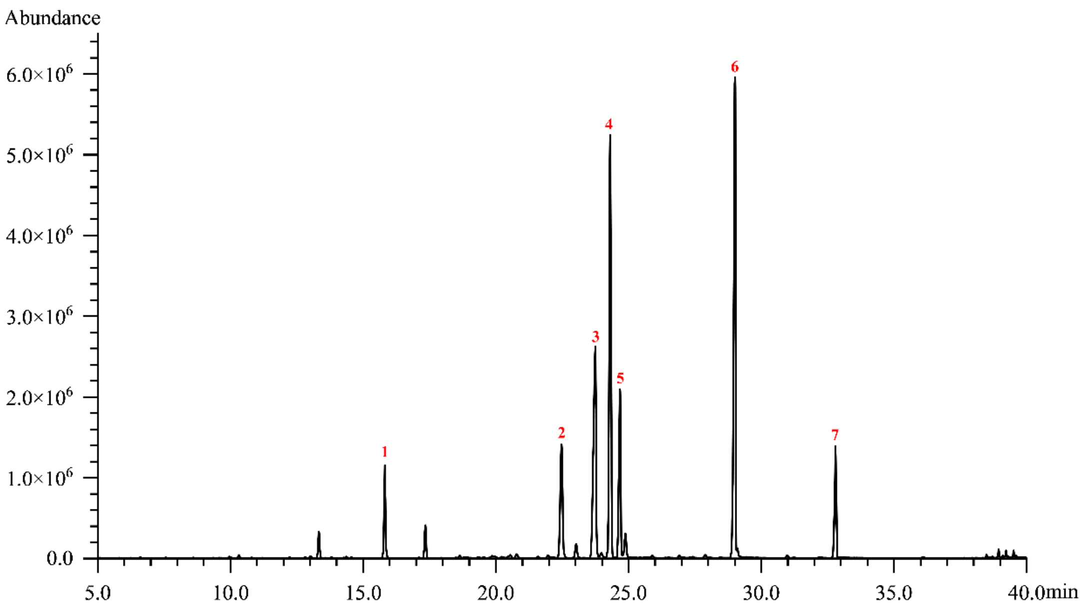

Fig. S4. GC chromatogram obtained for sample 500Cr

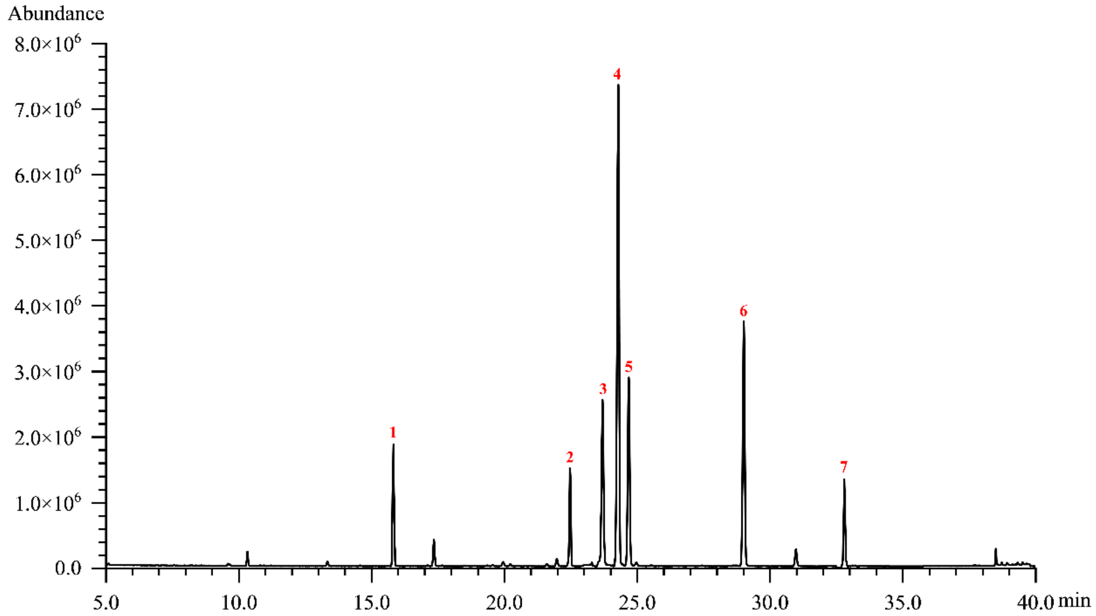

Fig. S5. GC chromatogram obtained for sample 1000Cr

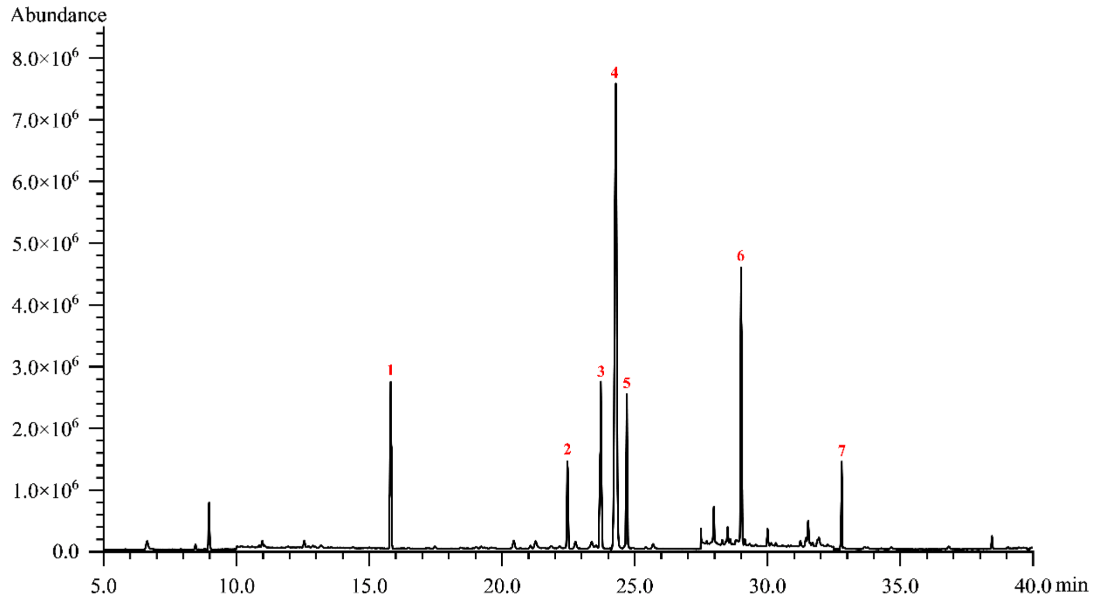

Fig. S6. GC chromatogram obtained for sample 1500Cr

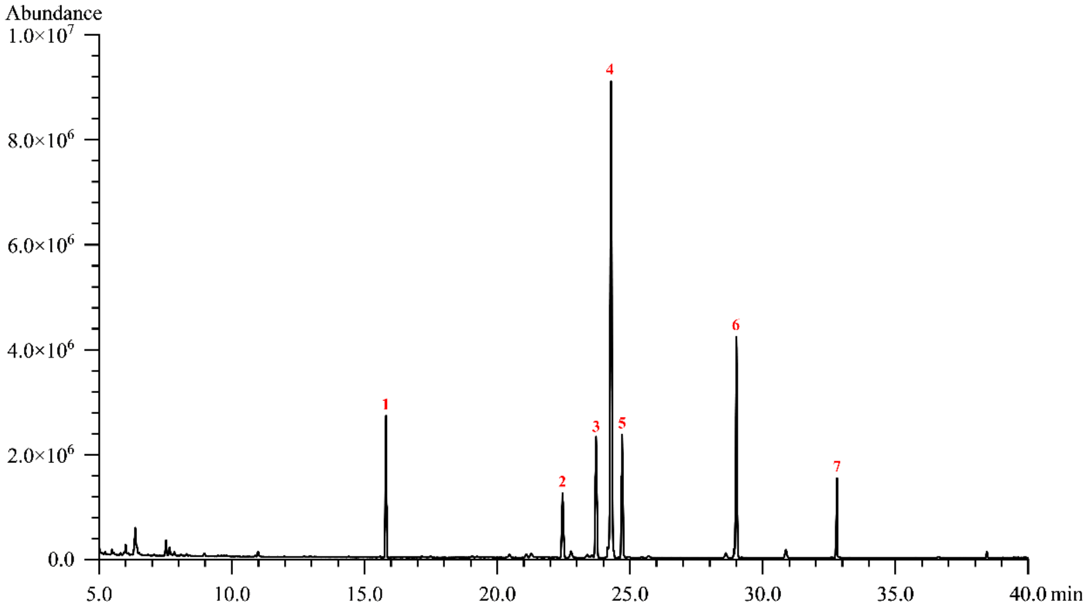

Fig. S7. GC chromatogram obtained for sample 3000Cr
